# Prospects for Using Phosphate-Solubilizing Microorganisms as Natural Fertilizers in Agriculture

**DOI:** 10.3390/plants11162119

**Published:** 2022-08-15

**Authors:** Anna Timofeeva, Maria Galyamova, Sergey Sedykh

**Affiliations:** 1 SB RAS Institute of Chemical Biology and Fundamental Medicine, 630090 Novosibirsk, Russia; 2 Faculty of Natural Sciences, Novosibirsk State University, 630090 Novosibirsk, Russia

**Keywords:** phosphate solubilization, soil microbiome, sustainable agriculture, biofertilizer, soil bacteria, nitrogen fixation, phosphate fertilizers

## Abstract

Phosphates are known to be essential for plant growth and development, with phosphorus compounds being involved in various physiological and biochemical reactions. Phosphates are known as one of the most important factors limiting crop yields. The problem of phosphorus deficiency in the soil has traditionally been solved by applying phosphate fertilizers. However, chemical phosphate fertilizers are considered ineffective compared to the organic fertilizers manure and compost. Therefore, increasing the bioavailability of phosphates for plants is one of the primary goals of sustainable agriculture. Phosphate-solubilizing soil microorganisms can make soil-insoluble phosphate bioavailable for plants through solubilization and mineralization. These microorganisms are currently in the focus of interest due to their advantages, such as environmental friendliness, low cost, and high biological efficiency. In this regard, the solubilization of phosphates by soil microorganisms holds strong potential in research, and inoculation of soils or crops with phosphate-solubilizing bacteria is a promising strategy to improve plant phosphate uptake. In this review, we analyze all the species of phosphate-solubilizing bacteria described in the literature to date. We discuss key mechanisms of solubilization of mineral phosphates and mineralization of organic phosphate-containing compounds: organic acids secreted by bacteria for the mobilization of insoluble inorganic phosphates, and the enzymes hydrolyzing phosphorus-containing organic compounds. We demonstrate that phosphate-solubilizing microorganisms have enormous potency as biofertilizers since they increase phosphorus bioavailability for the plant, promote sustainable agriculture, improve soil fertility, and raise crop yields. The use of phosphate-solubilizing microbes is regarded as a new frontier in increasing plant productivity.

## 1. Introduction

Phosphorus (P) is one of the most important macronutrients for the growth and development of plants [1,2]. It comprises 0.2 to 0.8% of the dry weight of plants [3] and is found in nucleic acids, enzymes, coenzymes, nucleotides, and phospholipids [4]. Phosphorus is the second most important macronutrient necessary for plants, after nitrogen. The average phosphorus content in soil is about 0.05% (by weight), with only 0.1% of this amount available to be used by plants [5]. Plant roots can absorb phosphorus in the form of orthophosphates H_2_PO_4_^−^ or HPO_4_^2–^, but the concentration of these ions in the soil is in the micromolar range [6]. In soil, most phosphates are present as insoluble iron, aluminum, and calcium phosphates.

Phosphates are involved in various physiological and biochemical reactions, including photosynthesis, root and stem development, flower and seed formation, crop maturation, nitrogen fixation in legumes, and resistance to plant diseases [3]. Phosphates are among the most important factors limiting crop yields [7].

The problem of phosphate deficiency in the soil is conventionally solved by using phosphorus fertilizers. However, with phosphate anions in chemical fertilizers being extremely reactive and quickly fixed due to interactions with Ca^2+^, Fe^3+^, and Al^3+^ in the soil, most of the phosphorus added to fertilizers is inaccessible to plants. Thus, the formation of insoluble complexes of corresponding phosphate salts leads to the efficiency of using chemical phosphate fertilizers of only 5–25% [8]. Also, the long-term application of phosphorus fertilizers causes soil acidification, water pollution, and eutrophication [9]. Therefore, increasing the bioavailability of soil-insoluble phosphate for the plants is one of the primary goals of agriculture and forestry development.

Soil microorganisms play a key role in plant nutrient uptake and are involved in a wide range of biological processes. Some of them can mineralize insoluble soil phosphorus, making them available for plant consumption. Aside from chemical fertilization, microbial phosphate solubilization and mineralization seem to be the only possible way to increase the amount of phosphorus accessible for plants. Numerous microorganisms in the soil and rhizosphere are known to effectively release phosphorus from the soil through solubilization and mineralization [10], and these are referred to as phosphate-solubilizing microorganisms.

Phosphate-solubilizing microorganisms are gaining attention due to their advantages, such as safety for the environment, low cost, and high efficiency [11,12]. The use of phosphate biofertilizers is a promising approach to improving food production and increasing crop yields. Phosphate-solubilizing microorganisms can promote plant growth by enhancing the efficiency of biological nitrogen fixation and phytohormone synthesis and increasing the availability of certain micronutrients, such as zinc and iron [13]. Phosphate-solubilizing microorganism inoculations have been proven to increase plant yield and phosphorus uptake both in pots and in the field [14].

Facilitating phosphate uptake and utilization by plants is critical for economic and environmental reasons. The solubilization of phosphates by soil microorganisms is regarded as a particularly prospective research object. Thus, the inoculation of soil or crops with phosphate-solubilizing microorganisms is considered a promising strategy for improving plant phosphorus uptake [15]. Here, we review the availability of soil phosphate, the diversity of phosphate-solubilizing soil microorganisms, the mechanisms and induction of phosphate solubilization and their role in plant growth, and the potential role of such microorganisms as natural biofertilizers in crop production.

## 2. Soil Phosphate-Solubilizing Microorganisms

Many types of soil bacteria and fungi can dissolve phosphates in vitro. Phosphate-solubilizing microorganisms increase the bioavailability of soil phosphorus for plants [5]. They dissolve insoluble inorganic (mineral) phosphorus and mineralize insoluble organic phosphates [3]. Salt-tolerant or halophilic soil microorganisms are able to dissolve insoluble phosphates, thereby contributing to the development of agriculture on saline and alkali soils [5].

The ability to solubilize phosphates was characterized in detail for mycorrhizal fungi [16] that are also proven to contribute to the crop nutrition by increasing the volume of rhizosphere and thus the volume of soil from which phosphates can be absorbed [17]. Other soil microorganisms are phosphate-solubilizing bacteria (PSB) that are generally associated with the plant rhizosphere [18]. Table 1 provides information on the genera of phosphate-solubilizing bacteria and fungi.

According to the literature data, PSBs account for 1–50% of the total microbial population, compared to the phosphate-solubilizing fungi accounting for 0.1–0.5%. Phosphate-solubilizing microorganisms are ubiquitous, with their species composition varying from soil to soil. Most phosphate-solubilizing microorganisms have been isolated from the rhizosphere of various plants, where they are metabolically active [37].

## 3. Biochemical Properties of the Soil Related to the Bioavailability of Phosphates

Soil phosphates exist both in inorganic and organic form. The inorganic (mineral) form of phosphate is represented by primary (apatites, strengite, and variscite) and secondary minerals of phosphorus (iron, aluminum, and calcium phosphates) [38]. The release of phosphate from these minerals is slow and regulated by several factors, particularly by soil pH [38,39]. Under acidic conditions, phosphates are adsorbed on Al/Fe oxides and hydroxides such as gibbsite and goethite. Under alkaline conditions, phosphates are precipitated with calcium. Additionally, phosphorus may be immobilized on the soil clay particles, a phenomenon strongly influenced by the type of ions adsorbed on the surface of clay minerals [40].

Soil microorganisms can immobilize phosphates from the soil. After absorption by microbial cells, phosphorus is incorporated into the cellular structures of microorganisms (e.g., nucleic acids, organic phosphorus esters, free inorganic phosphate, and coenzymes), with excess phosphorus likely to be stored as polyphosphates [41]. Microbial biomass is an important temporary immobilized phosphorus pool that can be mineralized and released into the soil solution as available phosphorus long-term [42,43].

In most microorganisms, the role of a phosphate reserve is played by inorganic polyphosphates, linear polymers of phosphoric acid containing between three and several hundred phosphate residues [44]. Many bacteria accumulate polyphosphates under unfavorable conditions [45]. For example, the accumulation of polyphosphates in *E. coli* cells occurs under conditions of amino acid deficiency [46]. Enzymes catalyzing the synthesis of polyphosphates are polyP kinases. The role of polyP as a phosphate reserve has been proven for many microorganisms belonging to different taxa, from archaea to fungi [45]. Polyphosphates are rich in “high energy” of anhydride bonds [47], and can be used as an energy source due to the Pi release [48].

Various enzymes are involved in the consumption of accumulated polyphosphates and the breakdown of phosphates outside the cell. Polyphosphate utilization and degradation is catalyzed by polypases, including exopolypase (PPX), and several polyphosphate-specific kinases, including polyP-glucokinase and polyP-fructokinase [45].

The biomass of soil microorganisms stores significant amounts of phosphorus, protecting it from plant adsorption [49,50]. During the biogeochemical recycling process, the phosphorus accumulated by bacteria and fungi is slowly released back into the soil and becomes available to plants, as evidenced by the correlation between phosphorus uptake by plants and phosphorus biomass in microorganisms [51].

**Figure 1:**
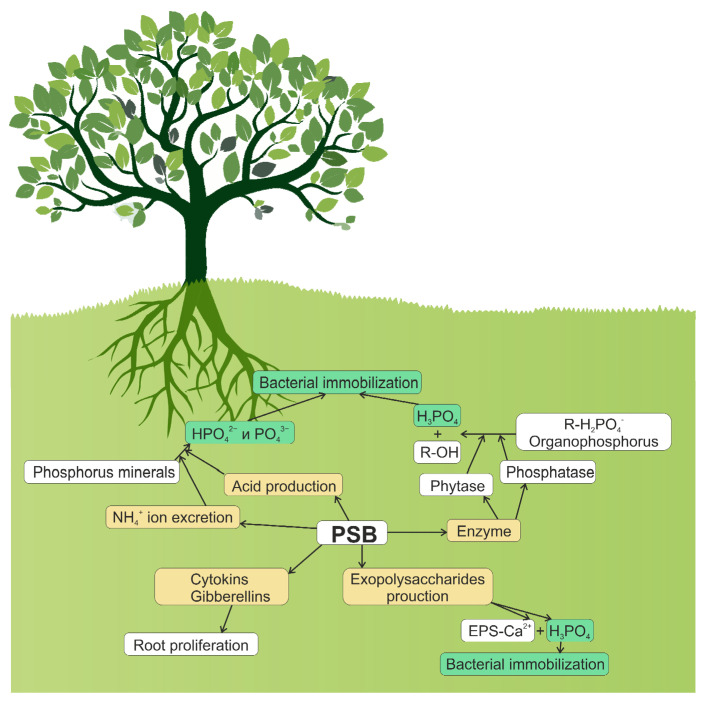
Role of PSBs in the solubilization of phosphates: solubilization of inorganic phosphates due to the synthesis of organic and inorganic acids; NH_4_^+^-excretion; solubilization of organic phosphorus-containing compounds due to the synthesis and excretion of enzymes; synthesis of polysaccharides, cytokinins, and gibberellins.

The rate of phosphate release from microbial biomass likely depends on the amount of phosphorus available in soil [52], carbon availability [53], soil texture [54], and composition of microbial community [55]. The ability of microorganisms to balance between solubilization (mineralization) and immobilization processes determines the extent to which these bacteria and fungi can improve the availability of phosphorus in the soil–plant system. Figure 1 depicts several biochemical routes of phosphate solubilization by soil bacteria: synthesis of organic and inorganic acids; excretion of ammonia ions; synthesis and excretion of phosphatases and phytases; and synthesis of polysaccharides, cytokinins, and gibberellins.

## 4. Mechanisms of Inorganic Phosphate Solubilization

In recent years, numerous studies have been done on the characteristics and mechanisms of phosphorus solubilization processes. PSBs are thought to be able to dissolve insoluble phosphates by secreting low molecular weight organic acids [56]. In alkaline soils, phosphates can precipitate to form calcium phosphates, their solubility increasing with a decrease in soil pH. PSBs increase phosphorus availability by secreting organic acids that cause a decrease in soil pH [57,58]. The increase of soil pH leads to the appearance of divalent and trivalent forms of inorganic phosphorus: HPO_4_^2−^ and PO_4_^3−^ [37].

Organic acids are produced in the periplasmic space by direct oxidation [59]. The release of these organic acids into the environment is accompanied by a decrease in pH. Surprisingly, there is no correlation between pH and the amount of solubilized phosphorus [60]. The mechanism of acidification was suggested: the release of H^+^ is associated with the assimilation of cations. For example, NH_4_^+^ assimilation together with H^+^ excretion leads to phosphate solubilization [61]. An alternative mechanism for the solubilization of mineral phosphates for producing organic acids is the release of H^+^ to the extracellular surface via the exchange of cation or the ATPase activity with H^+^ translocation [18]. Also, assimilation of NH_4_^+^ in microbial cells was reported to be accompanied by proton release, leading to the solubilization of phosphorus without any organic acids being formed [3].

The efficiency of solubilization depends on the strength and nature of the acids. Tri- and dicarboxylic acids are more efficient than monobasic and aromatic acids, and aliphatic acids are more efficient in phosphate solubilization than phenolic, citric, and fumaric acids. Among the organic acids that solubilize phosphates are primarily citric, lactic, glicolic, 2-ketogluconic, oxalic, glyconic, acetic, malic, fumaric, succinic, tartaric, malonic, glutaric, propionic, butyric, glyoxalic, and adipic acids [37], with gluconic acid and 2-ketogluconic acid being the most common solubilizing agents for mineral phosphates. Listed in Table 2 are organic acids synthesized by PSBs.

Organic acids are synthetized as products of carbon metabolism [79,80], which is closely related to the concentration of soluble phosphate [81]. The production of acids such as gluconic acid and pyruvic acid by PSB was proven to be significantly affected by the concentration of soluble phosphate [82,83]. The conversion of glucose to the organic acids by the cells of PSBs in phosphate deficient conditions is higher than in phosphate sufficient conditions, and it is suggested that the effect of soluble phosphate on organic acid production underlies the regulation of soluble phosphate in PSB mediated mineral phosphate solubilization [82].

Low-molecular-weight organic acids are synthetized during glucose oxidation through direct periplasmic oxidation [79] and intracellular phosphorylation [80]. In the *P. aeruginosa P4* strain, phosphate deficiency contributes to an increase in gluconic acid secretion, which is caused by the change of the predominant glucose metabolism pathway from phosphorylation to direct oxidation [82].

Gluconic acid was proven to be an effective organic acid component in mineral phosphate solubilization in *Serratia marcescens* [84], and *E. herbicola* [85]. *B. multivorans* WS-FJ9 solubilizes the phosphate in SP medium using only gluconic and pyruvic acids. However, gluconic acid production is not affected by changes in the concentration of soluble phosphate [86], which could be due to the constitutive nature of the direct oxidative pathway of glucose metabolism [82]. Soil fungi may be even more important than bacteria since they typically produce and excrete more acids, such as gluconic, citric, lactic, 2-ketogluconic, oxalic, tartaric, and acetic acid [3]. Other mechanisms of mineral phosphate solubilization by microorganisms are the production of inorganic acids (sulfuric, nitric, and carbonic acids) and the secretion of chelating agents. However, the effectiveness of inorganic acids and chelating agents in the phosphorus release into the soil is lower than that of organic acids [68]. Another mechanism of microbial phosphate solubilization was reported to be the secretion of enzymes [5].

## 5. Mineralization of Organic Phosphate-Containing Compounds

The content of organic phosphorus in the soil can reach 30–50% of the total amount, with soil organic phosphorus being found primarily in the form of inositol phosphate (soil phytate). Other organic phosphorus compounds were reported, including: phosphomonoesters, phosphodiesters, phospholipids, nucleic acids, and phosphotriesters [18]. Additionally, large quantities of xenobiotics (pesticides, detergents, antibiotics, and flame retardants) that are regularly released into the environment are also known to contain organic P. Most of these organic compounds are of high molecular weight and resistant to chemical hydrolysis. For plant uptake, these compounds must be converted into soluble ionic phosphate (Pi, HPO_4_^2−^, H_2_PO_4_^−^) or low molecular weight organic phosphates [87].

Several groups of enzymes secreted by phosphate-solubilizing microorganisms are involved in the process of phosphate mineralization. The enzymes of the first group dephosphorylate phosphor-ester or phosphoanhydride bonds in organic compounds. These are non-specific acid phosphatases (NSAP). The most studied NSAP enzymes are phosphomonoesterases, also called phosphatases [88]. These enzymes can be acidic or alkaline phosphomonoesterases [89]. The pH of the soils possessing phosphatase activity was indicated to be from acidic to neutral, indicating that acid phosphatases play a major role in this process [18].

Phytase is another phosphate-solubilizing enzyme involved in the mineralization of organic P. This enzyme is responsible for releasing phosphorus from organic compounds in the soil (plant seeds and pollen) that are stored in the form of phytate (inositol polyphosphate). Phytase releases phosphates in a form that is available to plants. While plants cannot obtain phosphorus directly from the phytate, the presence of phosphate-solubilizing microbes in the rhizosphere can compensate for the inability of plants to obtain phosphates directly from phytate [43]. Phytates are synthesized by plants and constitute a significant amount (from 60 to 80%) of organic phosphorus in the soil [90]. However, the ester bonds in phytic acid are quite stable and their natural degradation is practically impossible [91]. Microbial mineralization of phytate by phytase plays an essential role in the process of phosphorus recycling. Phytate can be completely hydrolyzed with the formation of one inositol and six molecules of inorganic phosphate, or partially with the formation of lower isomers of inositol polyphosphate and inorganic phosphates [92].

Among the four types of phytases identified, β-propeller phytase (BPP, EC 3.1.3.8 or EC 3.1.3.26) differs from the other three phytases (histidine acid phosphatase, cysteine phytase, and purple acid phosphatase) in that it has a neutral ∼pH 7.0 rather than an acidic pH optimum. It has been indicated that BPP is the main class of phytate degrading enzymes in nature [90,93]. A typical BPP has a six-lobed propeller fold with two phosphate-binding sites (a cleavage site and an affinity site) and six calcium-binding sites, three of which are high-affinity binding sites responsible for enzyme stability and three of which are low-affinity sites, regulating the catalytic activity of the enzyme [94,95].

Thus far, only a small number of BPP have been isolated and studied, including *Shewanella oneidensis* MR-1 PhyS [96], *Bacillus subtilis* PhyC [97], *Bacillus* sp. DS11 Phy [98], *B. subtilis* 168 168PhyA [99], *Bacillus licheniformis* PhyL [99], *Pedobacterobsis* 5 MJ11 PhyP [100], and *Janthinobacterium* sp. TN115 PhyA115 [101], all of which are mesophilic or thermophilic.

Phosphate solubilization by acid phosphatases was reported for *Pseudomonas* sp. [102], *Burkholderia cepacia* [103], *Enterobacter aerogenes*, *E. cloacae*, *Citrobacter freundi*, *Proteus mirabalis*, and *Serratia marcenscens* [104]. Moreover, solubilization of organic phosphate by phytase activity was observed in *Bacillus subtilis*, *Pseudomonas putida*, and *P. mendocina* [105], and phosphatase activity was discovered in *Klebsiella aerogenes* [106] and *P. fluorescens* [107]. However, although *P. sonchi* SBR5 possesses some of these enzymes associated with phosphate solubilization, the activation of the corresponding genes was not observed when differential gene expression was analyzed under the phosphate solubilization conditions [108].

The production of phosphatases by the soil microbiome was proved to be tightly controlled by the availability of inorganic phosphorus and nitrogen. The addition of nitrogen increases the phosphatase activity [109,110], while the supply of inorganic phosphorus suppresses the production and activity of phosphatases due to the negative feedback mechanism [110].

Phytase-producing fungi are *Aspergillus candidus*, *A. fumigatus*, *A. niger*, *A. parasiticus*, *A. rugulosus*, *A. terreus*, *Penicillium rubrum*, *P. simplicissimum*, *Pseudeurotium zonatum, Trichoderma harzianum, and Trichoderma viride*. Soil *Bacillus* and *Streptomyces* spp. are able to mineralize complex organic phosphates by producing extracellular enzymes such as phosphoesterases, phosphodiesterases, phytases, and phospholipases [37].

## 6. Bacterial Influence on the Root System

Auxin, ethylene, abscisic acid, cytokinins, and gibberellins are well-known phytohormones. PSBs can mediate the changes in the plant root system through the prevention of signaling and auxin transport. For example, *Phyllobacterium brassicacearum* (STM196) causes changes in the distribution of auxin in the root of *Arabidopsis* [111].

PSB can also cause changes in plant roots by supplying them with a significant amount of auxin that will act synergistically with endogenous auxin [112] and alter the development of the plant root system [113]. Bacterial production of phytohormones such as auxins and cytokinins can influence cell proliferation, leading to changes in root system architecture through overproduction of lateral roots and root hairs, followed by increased nutrient and water uptake [114]. An increase in root area is associated with an increase in symbiotic interactions with PSB, and thus an increase in phosphorus mobilization. Increased auxin content and higher plant biomass were reported for wheat inoculated with *Paenibacillus illinoisensis* (IB 1087) and *Pseudomonas extremaustralis* (IB-Ki-13-1). Thereby, auxin is discovered to play an important role in stimulating root growth and nutrient uptake [113]. Similarly, in corn and sugar cane, inoculation with *Paenibacillus* sp. and *Enterobacter cloacae* led to increased biomass of both above-ground and below-ground plant parts [115].

The enzymes produced by PSBs, such as 1-aminocyclopropane-1-carboxylate deaminase, were indicated to contribute indirectly to architectural and functional root modifications triggered by the substrate cleavage [116]. For example, the root biomass of rice plants was strongly correlated with the level of deaminase produced by *Alcaligenes* sp. [117].

## 7. Factors Affecting the Microbial Phosphate Solubilization

The ability of phosphate-solubilizing microorganisms to convert insoluble organic and inorganic phosphates is related to the properties of soil. Phosphate-solubilizing microorganisms of the soils under extreme environmental conditions, such as saline-alkaline soils, nutrient-deficient soils or soils from extreme temperature environments, tend to solubilize more phosphate than phosphate-solubilizing microorganisms of soils under temperate conditions [5]. There are conflicting reports on the effect of temperature on phosphate solubilization, with some authors specifying the optimal solubilization temperature of 20–25 °C [118], some indicating 28 °C [119], and the others suggesting 30 °C [120]. The ability of phosphate solubilization was demonstrated at an extreme temperature of 45 °C in desert soil [121,122] and at a low temperature of 10 °C [123].

Other factors influencing the microbial phosphate solubilization include the interaction with other soil microorganisms, the stage of plant vegetation, environmental conditions, types of soils of the climatic zone, plant types, agronomic practices, land use systems, and physico-chemical properties of the soil, including the amount of organic matter and pH [124]. Well-aerated soil is more conducive to phosphate solubilization compared to water-saturated moist soil. Among the phosphate-solubilizing bacteria, both anaerobic and aerobic are described. Bacteria of genera *Agrobacterium*, *Bacillus*, *Burkholderia*, *Micrococcus*, *Pseudomonas* are aerobic, genera *Aeromonas*, *Enterobacter*, *Erwinia*, *Paenibacillus*, *Serratia* are anaerobic of facultative anaerobic.

Adding a small amount of inorganic phosphate to the rhizosphere was indicated to stimulate the mineralization of phytic acid by bacteria and thereby improve the phosphorus nutrition of plants [125]. Also, it was reported that the physiological activity of PSBs is also affected by exogenous soluble phosphate: the growth rate of microorganisms depends on the level of available soluble phosphate [81]. The growth rate of the phosphate-solubilizing strain of *Pseudomonas aeruginosa* with an excess of phosphate was 25 times higher than in phosphate deficiency case [82]. The strain WS-FJ9 of *B. multivorans* grew better at high concentrations of soluble phosphate both in Petri dishes and in broth cultures [86], with the same results obtained for the *P. aeruginosa* P4 [82]. Phosphate-solubilizing activity of the *B. multivorans* WS-FJ9 strain was found to be significantly suppressed at a concentration of soluble phosphate of 5 mM and completely suppressed at a concentration of 20 mM [86] by a negative feedback mechanism [83]. Sensitivity to the soluble phosphate is found to be a major barrier to the widespread use of PSBs [86].

## 8. Solubilization of Mineral Phosphates: Molecular Genetics

The molecular genetics basis of mineral phosphate solubilization is not fully understood. With the production of organic acids considered to be the primary mechanism of mineral phosphate solubilization, it is believed that any gene involved in the synthesis of organic acids can influence the solubilization process [18].

It was indicated that the expression of pyrroloquinoline quinone synthase of *Erwinia herbicola* leads to the secretion of acid and solubilization of mineral phosphates [85]. This enzyme directs the synthesis of pyrroloquinoline quinone, a cofactor necessary for the formation of glucose dehydrogenase holoenzyme, which catalyzes the formation of gluconic acid from glucose by direct oxidation.

Similarly, the gene of mineral phosphate solubilization (*gabY*) was isolated in *Pseudomonas cepacia* [126]. Expression of the *gabY* gene allows the mineral phosphate solubilization phenotype to be induced through the production of gluconic acid. The *gabY* gene did not indicate a clear homology with the pyrroloquinoline quinone synthetase gene [85]. The *gabY* gene may play an alternative role in the expression and/or regulation of the direct oxidation pathway in *Pseudomonas cepacia* by acting as a functional in vivo mineral phosphate-solubilization gene. Thus far, very little is known about the genetic regulation of mineral phosphate-solubilization processes [127].

Most strains of *Paenibacillus* can solubilize phosphate through the production of gluconic acid. A study of 35 strains, including at least 18 species, indicated all but two strains to contain genes involved in the production of gluconic acid, encoding glucose-1-dehydrogenase and gluconic acid dehydrogenase. Strains apparently lacking these genes are *P. beijingensis* and *P. terrae* HPL-003. Strains carrying such genes include *P. azotofixans*, *P. curdlanolyticus*, *P. dendritiformis*, *P. elgii*, *P. forsythia*, *P. graminis*, *P. lactis*, *P. massiliensis*, *P. mucilaginosus*, *P. peoriae*, *P. polymyxa*, *P. sabinae*, *P. sonchi*, *P. sophorae*, *P. vortex*, and *P. zanthoxyli*. All of the genomes analyzed were found to contain genes for uptake and degradation of phosphonates, containing a highly stable C–P bond, as well as genes for a phosphate-specific transport system [128]. Phosphate solubilization was confirmed for *P. elgii*, *P. kribbensis*, *P. macerans*, *P. mucilaginosus*, *P. polymyxa*, *P. xylanilyticus* and several unclassified strains [129].

### 8.1. Mineralization of Organic Phosphate: Molecular Genetics

Different patterns of phosphatase activity are widespread in bacteria, especially in *Enterobacteriaceae*. Phosphatase production is often controlled by complex regulatory mechanisms, resulting in enzyme activity being detected only under certain environmental conditions. However, there is still a lack of full understanding of the properties, regulation, and role of these enzymes. Even in the most thoroughly studied bacteria, *Escherichia coli* and *Salmonella typhimurium*, only a few genes have been cloned and studied for their effect on the regulation of phosphatase activity [18].

The primary factor influencing the microbial production of phosphatases is the concentration of inorganic phosphate. This mechanism is best understood for *E. coli* alkaline phosphatase (*PhoA*) that is suddenly and completely induced when the inorganic phosphate concentration decreases from 100 mM to 0.16 mM [130]. The gene network includes the phosphate transport operon as a regulatory element, in addition to the sensor-activator operon. The regulation is due to the genes controlled by inorganic phosphate concentration and is activated by *PhoB* [18].

Another bacterial phosphatase repressed by inorganic phosphate is alkaline phosphatase from *Morganella morganii,* which is produced in conditions of low inorganic phosphate availability. This alkaline phosphatase is likely similar in its regulation and molecular weight of its polypeptide components to that in *E. coli* [131]. *Pseudomonas fluorescens* MF3, *Providencia stuartii* and *P. rettgeri* also have alkaline phosphatase activity that is also inhibited by inorganic phosphates [104]. Some studies suggest that the regulation of phosphatase gene expression in other families, such as those belonging to the *Enterobacteriaceae* family, may be like the *E. coli* pho genes. These data are based on a high degree of homology of the promotor structures between these genes [132].

Production of PhoN enzyme (acid phosphatase of class A) of *Salmonella enterica* serovar *typhimurium* is moderately induced by inorganic phosphate starvation [133]. This gene is under the control of the two-component regulatory system *PhoP*–*PhoQ*, promoting the transcription of *PhoN* and other *PhoP* genes that are activated at low concentrations of Mg^2+^ in the environment [134].

Most of the alkaline phosphatases found in the family *Enterobacteriaceae* are repressed by inorganic phosphate, while many acid phosphatases are unrepressed with phosphates. For some bacterial phosphatases, other regulatory systems were described. For example, the expression of the apo gene encoding the acid phosphatase enzyme in *Pseudomonas fluorescens* MF3 was determined to be regulated by temperature [135].

The literature data indicate that the regulation of phosphatases is a complex system requiring considerable additional research. In any case, the existing knowledge about bacterial phosphatases provides the basis for better understanding and further investigation of the phosphatase expression in soil bacteria [18].

Nonspecific acid phosphatases (phosphohydrolases or NSAP) released by phosphate-solubilizing microorganisms and dephosphorylating phosphodiester or phosphoanhydride bonds in P-containing substances play an important role in the mineralization of phosphates in most soils [136]. These NSAPs can be both acidic or alkaline phosphomonoesterases [137]. Several NSAP genes were isolated and characterized [136]. For example, the *olpA* gene of *Chryseobacterium meningosepticum* encodes a wide range of phosphatases that efficiently hydrolyze monophosphates and phosphorylated sugars [138]. *PhoN* gene of *Deinococcus radiodurans* and *PhoK* gene of *E. coli* are able to encode periplasmic acid phosphatase and extracellular alkaline phosphatase to enhance the biomineralization of toxic ions in contaminated soils [139]. The cyanobacteria *Microcystis aeruginosa* contain genes encoding an extracellular alkaline phosphatase that solubilized various inorganic and/or organic phosphates [140]. The phytase enzyme encoded by the *appA* and *phyA* genes is another important enzyme for phosphate mineralization responsible for phosphorus release from phytate in the soil [137].

The transcriptome analysis of the phosphate-solubilizing strain of *B. multivorans* WS-FJ9 isolated from the rhizosphere of *Pinus elliotii* Engelm indicated the global activation of genes involved in the growth of WS-FJ9 stimulated by high concentrations of soluble phosphate. Among these genes, there are genes encoding the as flagellar MS-ring protein, flagellar hook protein FlgE, and Fe-S protein HscA. However, the phosphorylating pathway of glucose metabolism in *B. multivorans* WS-FJ9 is also affected by soluble phosphate. The expression of the glycerate kinase gene and the 2-oxoglutarate dehydrogenase gene increased continuously with a decrease in soluble phosphate; phosphate deficiency contributed to the activation of these genes [86]. Glycerate kinase and 2-oxoglutarate dehydrogenase are rate-limiting enzymes in both the Entner–Doudoroff and the tricarboxylic acid cycle pathways [141]. These two pathways of glucose metabolism are successive ones of the phosphorylating pathway [142]. Additionally, pyruvate generated in the glycolysis pathway was found in *B. multivorans* WS-FJ9, and its level was inversely proportional corresponding to the concentration of soluble phosphate, indicating the repression of the phosphorylating pathway by soluble phosphate [86].

In the *Pseudomonas putida* CSV86 strain, the phosphorylation pathway was found to be regulated by the glucose metabolizing enzymes and/or the glucose transport process [143]. Genes involved in the ABC-type sugar transporter system were overexpressed in phosphate deficient *B. multivorans* WS-FJ9 [86], which was considered an important transport system for glucose uptake and transport [144]. Therefore, soluble phosphate was found to negatively regulate the glucose phosphorylation pathway in *B. multivorans* WS-FJ9, which may affect the organic acid secretion and, consequently, phosphate-solubilization activity.

Additionally, the gene of the histidine protein kinase *PhoR* was found to be activated by the low concentration of soluble phosphate [86]. *PhoR* is considered an important part of the two-component regulatory operon PhoB–PhoR (Pho Regulon) [145]. This two-component transcription factor regulates the expression of several Pho regulon genes, such as the *PhoA* and *PhoB* alkaline phosphatase genes [146] and the gene of APase-alkaline diphosphoesterase *PhoD* [147] encoding enzymes involved in the mineralization of organic phosphates.

In addition to the genes involved in phosphate solubilization, the expression levels of many transcriptional regulatory genes and genes involved in signal transduction are regulated at various concentrations of soluble phosphate. The transcription regulator of the LysR family negatively regulates the set of genes, including those involved in bacterial virulence, metabolism, and motility [148]. TonB-dependent regulatory systems perceive signals outside the bacterial cell and transmit them to the cytoplasm, where the TonB-dependent receptor is involved in both transport [149] and signal conversion [150]. The expression levels of these genes are regulated by soluble phosphate. These genes both directly and indirectly contribute to the response to exogenous soluble phosphate.

The transcriptome analysis revealed a complex response of *B. multivorans* WS-FJ9 to the various levels of exogenous soluble phosphate. This finding may help in understanding the molecular mechanism behind the regulation of soluble phosphate on the physiological activity of PSB, especially on phosphate solubilization. This knowledge is expected to provide the basis for a molecular engineering strategy to reduce the sensitivity of PSB strains to soluble phosphate, which could improve their relevant biological activity.

### 8.2. Regulation of Inorganic Phosphate in Bacterial Cells

The phosphate (Pho) regulon is a global regulatory mechanism involved in the control of bacterial inorganic phosphate, which was first described for *Escherichia coli* and later for many other bacterial species [151]. Pho-regulon activates extracellular enzymes generating inorganic phosphate from organic ones, phosphate-specific carriers, and enzymes involved in phosphate storage and preservation. The most conserved member of the Pho regulon in the bacteria is the Pi-specific transporter (Pst) [152]. The most common enzymes induced in response to inorganic phosphate starvation in bacteria are alkaline phosphatases (PhoA), phospholipases (PhoD), glycerophosphodiester phosphodiesterases, phytases, and 5’-nucleotidase [151]. To store inorganic phosphates, most bacteria induce the expression of polyphosphate kinases, which are able to accumulate polyphosphate and, if necessary, reuse it [153]. To conserve nutrients, some bacteria can replace teichoic acids that are rich in inorganic phosphate polymers found in the cell wall of Gram-positive bacteria with teichuronic acids not containing phosphates [154].

The Pho regulon is controlled by a two-component regulatory system that includes an inner membrane histidine kinase sensor protein and a regulator of the cytoplasmic transcriptional response. These proteins were named differently in some bacteria, for example, PhoR–PhoB in *E. coli* [155], PhoR–PhoP in *Bacillus subtilis* [156], PnpR–PnpS in *Streptococcus pneumoniae* [157], etc. In all cases of inorganic phosphate deficiency, the response regulator is phosphorylated at the aspartic acid residue by the sensor kinase. The phosphorylated response regulator can bind to the specific DNA sequences and activate or inhibit gene transcription. These specific sequences, known as PHO boxes, were first characterized in *E. coli* [158] as the sum of two 11-nt direct repeat units, each consisting of seven highly-conserved and four less-conserved nucleotides [159]. Similar features were observed in many other bacteria, including *B. subtilis* [156], *Sinorhizobium meliloti* [160], *Corynebacterium glutamicum*, and *Streptomyces coelicolor* [10.1128/JB.00121-07]. The consensus sequence of the PHO box is found to significantly vary between bacteria [159].

The depletion of inorganic phosphate in the medium is important for the activation of the Pho regulon in bacteria [161]. In *E. coli*, in addition to the two-component PhoR–PhoB system, the inorganic phosphate uptake pathway requires five more proteins, four of them being the components of the Pst and one a component of the PhoU metal-binding protein. When concentration of inorganic phosphate is limited, PhoB is activated by PhoR acting as a kinase, but under conditions of excess free phosphate, PhoB activation is interrupted by PhoR acting as a phosphatase [162]. PhoU is required for PhoB dephosphorylation under phosphate-rich conditions [163].

Although *PhoU* is found in many bacterial genomes, this gene is absent in *B. subtilis*. The inorganic phosphate-signaling network in this bacterium includes a positive feedback loop between the PhoP-PhoR and ResD-ResE two-component systems [164]. ResD does not bind to the phoPR operon and appears to transfer its control through the expression of terminal oxidases [165].

### 8.3. Interaction of PSB with the Plant Roots

Most plants express two types of inorganic phosphate transporters to maintain intracellular phosphate concentrations required for optimal cell function. These genes (PHT) are divided into two families: (1) high-affinity *PHT1* transporters expressed in the roots and (2) low-affinity *PHT2* transporters responsible for the intracellular transport of phosphates in plant shoots [166]. Several PSB species have been identified as regulating the expression of specific plant genes. *PHT1* gene expression in plant roots has been found to be modulated by *Pseudomonas putida* (MTCC 5279) which is known to increase the expression of *PT1*, the member of the *PHT1* gene family in *Arabidopsis thaliana* roots during phosphate deficiency and salt stress [167]. Under the combined stress and in the presence of *P. putida*, the expression of *PT2* also increased. However, the expression of *PHO2* (the gene responsible for the accumulation of phosphate in shoots) was suppressed due to the *PHO2* gene playing an important role in phosphate-signaling under low phosphate conditions.

Similarly, increased phosphorus uptake in wheat inoculated with a consortium of *Bacillus species* was found to result from increased expression of the *PHT1* transporter gene in the roots [168]. Thus, rhizospheric PSB can trigger the changes in phosphate transporter gene expression either directly by altering plant metabolism associated with phytohormone production or indirectly by increasing phosphate availability. These molecular changes in roots (up- and down-regulation of phosphate transporters) have now been partially proven to be induced by PSB, positively influencing the intake of phosphate and subsequently reflecting a positive effect on plant growth and yield.

## 9. PSB Co-Inoculation of Plants and PSB Synergy with the Use of Phosphate Fertilizers

Inoculation with a mixture of PSBs belonging to *Bacillus*, *Pseudomonas*, and *Streptomyces* genera demonstrated better results in terms of plant growth and establishment than a single inoculation. Co-inoculation of wheat with phosphate-solubilizing and auxin-producing rhizobacteria *Pseudomonas fluorescens* Ms-01 and *Azosprillum brasilense* DSM1690 increased the biomass of roots and shoots compared with a single inoculation with the same bacteria [169]. Similarly, inoculation of amaranth plants with a consortium of three bacteria [*Bacillus firmus* (KUCr1), *Cellulosimicrobium cellulans* (KUCr3), and *Pseudomonas aeruginosa* (KUCd1)] increased the total root length and overall plant growth compared to mono- and double inoculation with different strains [170].

The studies indicate that the synergism of combining PSBs and phosphate fertilizers may increase the agronomic efficiency of the fertilizers in soils [171,172]. For example, maize growth was greatly improved by PSB inoculation in combination with various types of inorganic and organic phosphate fertilizers: livestock manure, bird droppings, single superphosphate, and rock phosphates [173]. The improvement of maize growth parameters was observed in response to inoculation with phosphate-solubilizing *Enterobacter sakazakii* J129 in combination with NPK fertilizer [174]. Also, increased phosphate uptake and utilization efficiency were reported for the wheat inoculated with *Pseudomonas* sp. or *Enterobacter* sp. along with the diammonium phosphate- based fertilizer [175].

The use of PSBs is indicating promise as a biotechnological intervention to improve the agronomic efficiency of rock phosphates as direct phosphate fertilizers. *Enterobacter* sp. was proven to be capable of increasing the solubilization of the phosphate from rock-phosphate up to 17.5% [176]. Also, PSBs combined with rock phosphate were proven to significantly increase the availability of phosphate in the soil (27%) compared to the release from rock phosphate alone (4%) [177]. A single inoculation of wheat plants with five different strains of *Pseudomonas* (*P. plecoglossicida, P. reinekei, P. koreensis*, *P. japonica*, and *P. frederiksbergensis*) in combination with rock-phosphate fertilizer was observed to increase phosphate bioavailability, nutrient uptake, chlorophyll content, morphological characteristics, and root structure associated with higher phosphate uptake compared to non-inoculated plants [178]. Moreover, using a consortium of three strains (*Pseudomonas corrugata* SP77, *P. koreensis* LT62 and *P. frederiksbergensis* G62) was also identified as having positive effects on plant growth, e.g., in enhancing the growth of *Medicago truncatula* when combined with rock-phosphate [179].

Not only are PSBs known to increase the agronomic efficiency of mineral phosphorus fertilizers, but also combinations of these bacteria with organic P-fertilizers have been reported. For example, the combined use of bioorganic phosphorus (a mixture of compost, biogas residues) and *Bacillus* sp. was found to increase the availability of phosphate in the soil and the PSB population [180]. Similarly, growth parameters of chili plants were proven to improve in response to PSB inoculation with simultaneous application of organic phosphate fertilizers (eggshell, tea powder, animal bone waste) [181]. Moreover, partial replacement of mineral fertilizers with organic fertilizers can increase the availability of phosphate in the soil and change the shape of the PSB community in the soil, leading to improved phosphate mineralization and its uptake by rice plants [182].

Thus, the inoculation of plants with PSBs in combination with mineral or organic fertilizers is regarded as a promising integrated strategy to increase the availability of phosphates by increasing the agronomic efficiency of using phosphate fertilizers to improve soil fertility and support agriculture.

### Symbiotic Interactions between Phosphate-Solubilizing and Nitrogen-Fixing Microorganisms

Some microorganisms at the soil–root interface establish the nitrogen-fixing symbiosis with legumes through the formation of nodules on roots or stems [183]. Biological nitrogen fixation is a symbiotic process [184] during which plants provide carbon as an energy source for symbiotic microorganisms in exchange for bacteria-derived nitrogen compounds that can be easily assimilated by the host plant [185]. Almost 80% of the nitrogen available to plants originates from a symbiotic group, including *Rhizobium* which invades plants to form nodules. The non-symbiotic group consists of free-living microorganisms, such as *Bacillus*, *Azotobacter*, *Azospirillum*, and *Herbaspirillum*, and others contributing approximately 5–10% of the biological input of nitrogen to the soil [186].

The effectiveness of the biological fixation of nitrogen in cereal legumes was proven to be significantly limited by phosphate availability [187]. A significant correlation between nodal phosphate content and nitrogen fixation was identified. An adequate supply of phosphates to plants was found to regulate several metabolic processes associated with nitrogen fixation, such as the assimilation of ammonium into amino acids and ureides [188] and the synthesis of mitochondrial and symbiosomal membranes for nitrogen-fixing nodules [189]. In general, the formation and functioning of nodules and the energy costs of nitrogen fixation in legumes are highly dependent on the phosphate status of plants and in nodule tissues, and inadequate phosphate status reduces the potential contribution of nitrogen fixation [171].

Nitrogen fixation, as one of the key biological processes of the rhizosphere, is known to correlate with phosphate availability, with phosphate deficiency severely limiting the activity of diazotrophic (nitrogen-fixing bacteria) and reducing the symbiotic partnership between the host plant and rhizobia, in addition to the nitrogen fixation process itself [190]. As a rule, phosphate deficiency impairs the formation, development, and function of nodules by reducing the energy available to support the metabolic activity of nodules [191]. Plants living in symbiosis with nitrogen-fixing bacteria have a higher phosphorus demand than plants that do not have such symbionts [192]. The increased phosphorus demand in plants is generally due to the phosphates required for the nitrogen fixation processes [188,193]. However, other studies suggest that the high phosphate requirement is due to the needs of nitrogen-fixing bacteria, whose phosphorus requirements were found to be higher than those of non-nitrogen-fixing bacteria [172]. The availability of various phospholipids (such as phosphatidylserine, phosphatidylcholine, and phosphatidylethanolamine) may be crucial in maintaining the symbiosis between legumes and rhizobia [194]. These compounds are required to create the correct rhizobia membrane architecture to effectively participate in BNF [195,196].

Therefore, introducing phosphate-solubilizing microbes to nutrient-deficient soils, where the nutrients are stored in biologically inaccessible forms, could help farmers improve crop yields and obtain economic benefits through the rational use of phosphate fertilizers and the increase of nitrogen fixation. For example, the combination of nitrogen-fixing *Rhizobium* and phosphate-solubilizing *Bacillus megatherium* was observed to increase the yield and quality of *Vicia faba* seeds and the yield, nodule biomass, shoot biomass, and nitrogen and phosphorus content of *Lablab purpureus* [197].

The synergy between soil phosphate-solubilizing bacteria and rhizobial strains of nodule legumes is an important rhizosphere process that is worth investigating. Several studies have reported that the application of phosphate-solubilizing microorganisms, including PSB, to the soil, individually or in the consortia, increased plant growth by solubilization of insoluble phosphates and subsequent increase of nitrogen fixation [196]. Phosphate-solubilizing *Serratia,* combined with nitrogen-fixing *Mesorhizobium ciceri* and arbuscular mycorrhiza fungus *Glomus fasciculatum,* increased the growth of cereal legumes [191]. Similarly, co-inoculation of wheat with a diazotrophic *Paenibacillus beijingensis* BJ-18) and phosphate-solubilizing *Paenibacillus* sp. B1 significantly increased plant growth and the content of phosphates and nitrogen in the plant (roots and shoots) and the soil [198]. Furthermore, the inoculation of chickpeas with nitrogen-fixing *Mesorhizobium ciceri*, phosphate-solubilizing fungus (Penicillium WF6), and/or PSB *Serratia* T1 resulted in increased availability and uptake of phosphates and positively affected nitrogen fixation [199].

According to several studies, phosphate solubilization may be one of the main mechanisms by which PSBs enhance nitrogen fixation [200]. Bioavailable phosphates positively affect the symbiosis of legumes and rhizobia. Legumes treated with biofertilizers containing consortia of three PSBs: *Pseudomonas* sp., *Burkholderia* sp., and *Enterobacter* sp. in combination with rock phosphate had increased phosphate content both in the soil and plants, providing favorable conditions for nodulation, which in turn increased the efficiency of the nitrogen fixation [171].

Despite numerous reports highlighting interactions between phosphate-solubilizing and nitrogen-fixing bacteria, the mechanisms underlying these associations are not yet fully understood.

## 10. Perspectives

To enhance the use of PSBs as effective and important components in sustainable soil management systems, more data in needed on the molecular mechanisms they use to increase the bioavailability of phosphates. Consumers pay attention to the health, quality, and nutritional value of agricultural products. Thus, applying phosphate-solubilizing microorganisms as biofertilizers is one option to increase food production without posing a health risk, while saving natural sources of phosphate fertilizers and developing sustainable agriculture. It is important for researchers to continue studying phosphate-solubilizing microorganisms and translate this knowledge into a form that can be easily used by farmers [137].

## 11. Conclusions

The papers published in the recent years indicate that the efficiency of using phosphates in agriculture could be improved by inoculation with phosphate-solubilizing bacteria, which increase the availability of phosphate without disturbing the biochemical composition of the soil. These potential biofertilizers are universal since they can be used for various crops and generally are not specific for plants. Conversely, the development of personalized, plant-specific consortia of phosphate-solubilizers is likely to increase productivity further. Inoculation of phosphate-solubilizing microorganisms into the soil appears to be an effective way to convert insoluble phosphate compounds into bioavailable forms, resulting in better plant growth, yield, and quality. In this review, we demonstrate that the bacteria of genera *Bacillus*, *Pseudomonas*, *Rhizobium*, *Aspergillus*, and *Penicillium* genera are regarded as the most effective phosphate-solubilizers for increasing the phosphate bioavailability in soil. PSBs cause immediate plant growth by providing phosphates in an easily absorbable form. Additionally, phosphate-solubilizing microorganisms support plant growth by increasing the efficiency of nitrogen fixation. Two main mechanisms increasing free phosphate in the soil can be distinguished: the mobilization of inorganic and organic phosphates. In general, the mobilization of inorganic phosphate is performed primarily due to the release of organic acids by bacteria, and enzymes released into the extracellular environment perform the mineralization of organic phosphates.

While most farmers rely on inorganic sources of phosphates with which to avoid nutrient deficiencies, significant amounts of these fertilizers are lost from the soil through various mechanisms and are unavailable for plant uptake. In legume cropping systems, phosphate deficiency can also lead to nitrogen deficiency and reduced crop yields. Since the PSB-based biofertilizers have indicated promising effects on plant growth and yield, we assume that the phosphate-solubilizing microorganisms may be potential substitutes for inorganic phosphate fertilizers as a method to meet plant requirements and consequently increase the yields in sustainable agriculture. Their application is an environmentally and economically sound approach.

In this review, we described the molecular mechanisms of use phosphate-solubilizing microorganisms as biofertilizers. Solubilization of inorganic phosphate in the soil is found to increase its bioavailability for the plant, thus using PSBs in the soil is to promote sustainable agriculture, improve soil fertility, and increase crop yields. We consider use of PSB as microbial inoculants as the new frontier for increasing plant productivity. This technology can contribute to low-cost farming systems and a cleaner environment.

## Figures and Tables

**Table 1:** Genera of soil phosphate-solubilizing bacteria and fungi.

Bacteria		Fungi	
Genera	Ref.	Genera	Ref.
*Aeromonas*	[19]	*Achrothcium*	[3,20]
*Agrobacterium*	[18]	*Alternaria*	[3,20]
*Azotobacter*	[21,22]	*Arthrobotrys*	[3,20]
*Bacillus*	[23]	*Aspergillus*	[24]
*Bradyrhizobium*	[25]	*Cephalosporium*	[3,20]
*Burkholderia*	[19,26]	*Chaetomium*	[3,20]
*Cyanobacteria*	[3]	*Cladosporium*	[3,20]
*Enterobacter*	[22]	*Cunninghamella*	[3,20]
*Erwinia*	[27]	*Curvularia*	[3,20]
*Kushneria*	[5]	*Fusarium*	[3,20]
*Micrococcus*	[28]	*Glomus*	[3,20]
*Paenibacillus*	[29]	*Helminthosporium*	[3,20]
*Pseudomonas*	[30,31]	*Micromonospora*	[3,20]
*Rhizobium*	[32,33]	*Phenomiocenspora*	[3,20]
*Rhodococcus*	[25]	*Phenomiocenspora*	[3,20]
*Salmonella*	[25]	*Phenomycylum*	[34]
*Serratia*	[35]	*Populospora*	[3,20]
*Sinomonas*	[25]	*Pythium*	[3,20]
*Thiobacillus*	[25]	*Rhizoctonia*	[3,20]
		*Rhizopus*	[3,20]
		*Saccharomyces*	[3,20]
		*Schizosaccharomyces*	[3,20]
		*Schwanniomyces*	[3,20]
		*Sclerotium*	[36]
		*Torula*	[24]
		*Trichoderma*	[24]
		*Yarrowia*	[3,20]

**Table 2:** Important phosphate-solubilizing microorganisms, their ecological niches, and organic acids produced.

Phosphate-Solubilizing Microorganism	Ecological Niche	Predominantly Produced Acids	Ref.
*Escherichia freundii*	Soil	Lactic	[62]
*Aspergillus niger, Penicillium* sp.	Soil	Citric, glycolic, succinic, gluconic, oxalic, lactic	[62]
*Bacillus megaterium, Pseudomonas* sp., *Bacillus subtilus*	Soil rizoshpere	Lactic, malic	[63]
*Arthrobacter* sp., *Bascillus* sp., *Bacillus firmus B-7650*	Wheat and cowpea rhizosphere	Lactic, citric	[64]
*Aspergillus* sp., *Penicillium* sp., *Chaetomium nigricolor*	Lateritic soil	Oxalic, succinic, citric, 2-ketogluconic	[65]
*A. japonicus, A. foetidus*	Indian rock phosphate	Oxalic, citric, gluconic, succinic, tartaric	[66]
*P. radicum*	Wheat rhizosphere	Gluconic	[67]
*Enterobacter agglomerans*	Wheat rhizosphere	Oxalic, citric	[68]
*Bacillus amyloliquefaciens, B. licheniformis, B. atrophaeus, Penibacillus macerans, Vibrio proteolyticus, Xanthobacter agilis, Enterobacter aerogenes, E. taylorae, E. asburiae, Kluyvera cryocrescens, Pseudomonas aeromonassens, Chrysler*	Mangrove	Lactic, itaconic, isovaleric, isobutyric, acetic	[69]
*Penicillium rugulosum*	Venezuelan phosphate rocks	Citric, gluconic acid	[70]
*Enterobacter intermedius*	Grass rhizosphere	2-ketogluconic	[71]
*Aspergillus flavus, A. niger, Penicillium canescens*	Wheat grains	Oxalic, citric, gluconic, succinic	[72]
*Pseudomonas fluorescens*	Oil palms rhizosphere	Citric, malic, tartaric, gluconic	[73]
*Aspergillus niger*	Tropical and subtropical soils	Gluconic, oxalic	[74]
*P. trivialis*	Rhizosphere of *Hippophae rhamnoides* (cold Howl and Spiti deserts, Trans-Himalayas)	Lactic, formic	[75]
*B. pumilus var.2; B. subtilis var.2; Actinomadura oligospora; Citrobacter* sp.	Giant cardon cactus (*P. pringlei*)	Gluconic, propionic, isovaleric, heptonic, caproic, isocaproic, formic, valeric, succinic, oxalic, oxaloacetic, malonic	[76]
*B. pumilus CHOO8A; B. fusiformis*	*Opuntia cholla*	gluconic, oxalic, 2-ketogluconic, lactic, succinic aid, formic, citric, malic	[76]
*Bacillus* sp. *SENDO 6 и*	*P. pringlei*	Gluconic, propionic, isovaleric, formic, succinic, lactic	[77]
*Pseudomonas putida M5TSA, Enterobacter sakazakii M2PFe и Bacillus megaterium M1PCa*	*Mammillaria fraileana* cactus	Gluconic, propionic, acetic, formic, succinic, lactic, oxalic	[78]

## Data Availability

Not applicable.
